# A novel *in ovo* model to study cancer metastasis using chicken embryos and GFP expressing cancer cells

**DOI:** 10.17305/bjbms.2019.4372

**Published:** 2020-02

**Authors:** Robin Augustine, Hashim Alhussain, Anwarul Hasan, Mohamed Badie Ahmed, Huseyin C. Yalcin, Ala-Eddin Al Moustafa

**Affiliations:** 1 Department of Mechanical and Industrial Engineering, College of Engineering, Qatar University, Doha, Qatar; 2 Biomedical Research Center (BRC), Qatar University, Doha, Qatar; 3 College of Medicine, Qatar University, Doha, Qatara

**Keywords:** Tumor invasion, *in ovo* models, chicken embryo model, metastasis, GFP cells

## Abstract

Cancer metastasis is the leading cause of cancer-related mortality worldwide. To date, several *in vitro* methodologies have been developed to understand the mechanisms of cancer metastasis and to screen various therapeutic agents against it. Nevertheless, mimicking an *in vivo* microenvironment *in vitro* is not possible; while *in vivo* experiments are complex, expensive and bound with several regulatory requirements. Herein, we report a novel *in ovo* model that relies on chicken embryo to investigate cancer cell invasion and metastasis to various organs of the body. In this model, we directly injected green fluorescent protein (GFP) expressing cancer cells to the heart of chicken embryo at 3 days of incubation, then monitored cell migration to various organs. To this end, we used a simple tissue processing technique to achieve rapid imaging and quantification of invasive cells. We were able to clearly observe the migration of GFP expressing cancer cells into various organs of chicken embryo. Organ specific variation in cell migration was also observed. Our new slide pressing based tissue processing technique improved the detectability of migrated cells. We herein demonstrate that the use of GFP expressing cancer cells allows easy detection and quantification of migrated cancer cells in the chicken embryo model, which minimizes the time and effort required in this types of studies compared to conventional histopathological analysis. In conclusion, our investigation provides a new cancer metastasis model that can be further improved to include more complex aspects, such as the use of multiple cell lines and anti-metastatic agents, thus opening new horizons in cancer biology and pharmaceutical research.

## INTRODUCTION

Cancer metastasis is deemed responsible for about 90% of cancer-associated deaths [1]. This process is defined by cancer cell migration from a primary site to distal organs and subsequent formation of secondary tumors [2]. Therefore, the development of therapeutic modalities that prevent tumor metastasis is needed to cope with this devastating disease [3]. During metastasis, cancer cells undergo various steps such as invasion, intravasation, survival in the circulation, extravasation, and proliferation within the tissues of a remote organ [4,5]. Thus, cancer cells have to overcome various barriers (e.g., the blood-brain barrier in the case of brain metastasis) [6], as well as diverse sets of conditions to be successful in reaching distal tissue and subsequent proliferation [7]. Nevertheless, understanding the biological mechanisms behind cancer metastasis in order to develop new therapeutic agents to target them remains a major challenge in cancer research [8]. Today, appropriate *in vitro* [9] and *in vivo* [10] biological models are needed for the identification and validation of anti-metastatic drugs [11,12].

In general, *in vitro* cell migration models are simpler compared with the more complex *in vivo* ones [9]; however, *in vitro* models cannot mimic the complex physiological environment that allows for the study of the various steps involved in metastasis, and which can only be achieved using *in vivo* models [13]. Nevertheless, *in vitro* cell migration assays are essential in cancer metastases research as they provide a controlled environment which enables the collection of quantitative and consistent data [14]. Some of these models include transwell cell migration, wound healing or scratch, fence, spheroid migration, cell exclusion zone, micro-carrier bead, capillary tube, capillary chamber, and colloidal particle assays as well as time-lapse cell tracking [15]. This large number of migration assays that comprise two-dimensional (2D) in addition to the more complex three-dimensional (3D) models were developed to fulfill the need to study different types of cells and answer a variety of aspects related to cancer development, since no single *in vitro* model is sufficient on its own [16]. For instance, an *in vitro* 3D model for human head and neck squamous cell carcinoma (HNSCC) cannot mimic the systemic impact of invasion *in vivo* [17]. As a result of this insufficiency of the *in vitro* models, despite their unquestionable importance in metastatic research, it is necessary to reexamine *in vitro* data using *in vivo* models. Nevertheless, *in vivo* procedures come with a new set of complications, ranging from administrative hurdles of obtaining ethical approval to the higher expenses of animal models [18]. In addition, *in vivo* experiments are time consuming, laborious, require high technical skills, and high level of hands-on experience. However, they remain the most realistic model and provide the closest microenvironment to the human physiology, which makes them essential and mandatory before the start of any clinical trial [19].

On the other hand, it has been demonstrated for several decades that tumor tissues can be cultured within the chorioallantoic membrane (CAM) surrounding the chicken embryo [20,21] to study cancer metastasis [22]. In this regard, a relatively recent report used the CAM model to study HNSCC progression [17]. Although these earlier investigations were inspirational and laid the ground for the present study; such methods still rely on time-consuming experimental procedures such as histopathological sectioning, polymerase chain reaction (PCR)-based assays, etc [23,24]. Moreover, rather than depositing cells on the CAM, direct injection of cancer cells into the embryo can be advantageous as it will ensure the presence of injected cells in the blood stream.

In addition to the advances of *in vitro* and *in vivo* cell invasion models, robust imaging tools are required to detect migrated cells [25,26]. Thus, recent progresses in cell labeling, microscopy, and imaging technologies facilitate cell tracking, however, they remain complex and time-consuming [27,28]. Of these methods, fluorescent imaging enables easy visualization and tracking of fluorescently labeled individual cells that migrate to various organs of an animal [29]. Although fluorescent tags are largely available today, photobleaching and loss of fluorescence with time are a big challenge for their *in vivo* applicability. Conversely, advances in genetic engineering techniques, such as transfection, allow the generation of cells that can express fluorescent molecules [30]. Green fluorescent protein (GFP) is a biomarker with a wide range of applications for monitoring biological processes, tracking cells, detecting transgenic expression, and quantification of migrated cells in metastasis models *in vitro* and *in vivo* [31].

Thus, given the large gap between *in vitro* and *in vivo* microenvironments that often affect the transfer of promising therapeutic results; the need for a new model that combines the flexibility and consistency of *in vitro* methods in more complex physiology is essential. Herein, we propose a relatively simple *in ovo* model using chicken embryos and GFP expressing cancer cells that can be considered as a bridge between the *in vitro* and *in vivo* models.

## MATERIALS AND METHODS

**FIGURE 1:**
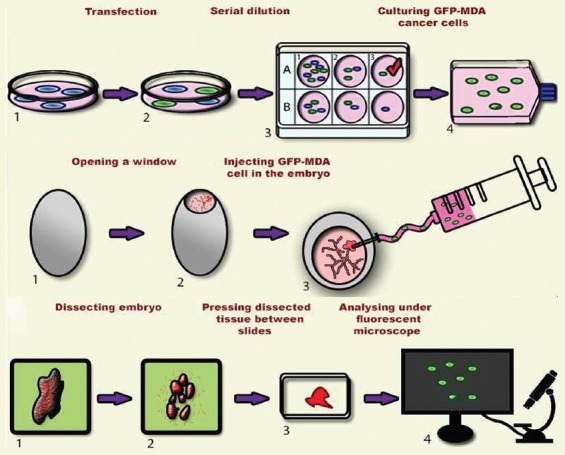
Schematic representation of the process flow of the developed metastasis models.

The general steps of the chicken embryo *in ovo* model for studying cancer cell metastasis are shown in Figure 1. These include: a) the generation of GFP expressing MDA-MB-231 (GFP-MDA) cells by plasmid-based transfection, b) injection of GFP-MDA-231 cells in chicken embryo, and c) dissection of the embryo after various time points, isolation of organs, microscopic slide preparation, and microscopic detection of cancer cell invasion. Detailed description of each step is given in subsequent sections.

### Selection criteria and culture of cell line

#### Selection criteria

In order to validate the proposed model, we used MDA-MB-231 cell line which is one of the most commonly used breast cancer cell lines in cancer research that was isolated from the metastatic mammary adenocarcinoma of a 51-year-old Caucasian female [32]. MDA-MB-231 is a highly aggressive, invasive, and poorly differentiated triple-negative breast cancer (TNBC) cell line with limited treatment options [33]. Studying the metastasis of TNBC is, therefore, crucial for finding novel treatment regimens.

#### Cell culture conditions

MDA-MB-231 cells are grown at 37°C in RPMI medium (Gibco, Thermo Fisher Scientific, US) supplemented with 10% fetal bovine serum (FBS) (Gibco) and 1% penicillin-streptomycin solution (Gibco). The cell line used in this study was at passage 37.

#### Generation of GFP expressing MDA MB-231 cells

Cells were cultured in a 24-well plate until they reached 70–90% confluence. Afterwards, cells were transfected using lipofectamine (Invitrogen, Thermo Fisher Scientific, US) and then serially diluted to obtain the clone of GFP positive MDA cells (GFP-MDA-231); the detailed protocol is listed below. After obtaining a stable cell line from the GFP positive cloned cells, they were diluted to the appropriate concentration for injecting in the embryo.

### Transfection procedure

Three hundred thousand MDA-MB-231 cells were seeded in 6-well plates and cultured in RPMI medium containing 10% FBS until 70–90% confluence. Upon reaching the required confluence stage, media were replaced with 2 ml fresh media (antibiotic-free). Then, 15 µg of the plasmid DNA was suspended in 1.5 ml of RPMI. Afterwards, 60 µl of Lipofectamine 2000 was mixed with another 1.5 ml RPMI. This was followed by an incubation period of 5 min at room temperature, then both solutions were combined and gently mixed. The mixture was incubated for 20 min at room temperature, after which, 500 µl of the above mixture was added to each one of the 6 wells and incubated at 37°C in a CO_2_ incubator for 6 h. Subsequent to the incubation period, the old media were replaced with 2 ml of fresh RPMI containing 10% FBS and incubated for 24 h in a CO_2_ incubator. Post 48 h of transfection, the GFP gene expression was visualized using a fluorescent microscope (Leica DMi8, Leica Microsystems, Germany) and the wells with the best transfection rates were marked (i.e., the well containing the highest percentage of fluorescent cells).

Stable GFP expressing MDA cell line (GFP-MDA-231) was established by single-cell cloning method using a reported protocol [34]. The selection process was performed in a 96-well plate by serially diluting 2 × 10^4^ cells/ml of the transfected GFP-MDA-231 cell suspension to get a single cell in some of the wells. The serially diluted 96-well plate containing the cells was incubated in a CO_2_ incubator for 48 h. Then, the plate was observed under the fluorescent microscope (Leica DMi8), and wells with only fluorescent cells were marked. Upon reaching 90–100% confluence, GFP-MDA cells were subcultured in a 12-well plate and eventually moved to larger tissue culture flasks.

### Injection of GFP-MDA-231 cells into the embryo

#### Incubation of the eggs

Fertilized chicken eggs were purchased from the Arab Qatari for Poultry Production and placed in an egg incubator at 37°C with 70% humidity. Thirty eggs were used for each set of experiment. Three independent sets of experiments were performed to get reproducible results. The rack turning cycle was set at 1 turn per hour. Eggs were not sprayed with 70% ethanol or any kind of liquid disinfectant as this can significantly reduce the survival rate, but wiped with a towel. The first day of incubation was considered as egg development day (EDD) zero (EDD-0). Injection procedure was performed at EDD-3.

#### Injection procedure

At EDD-3, the surface of the eggshell was disinfected with minimum amount of 70% ethanol and a very small circular window (4–6 mm) was made on the top blunt surface of the egg where the air sac was located and gradually widened (1.5–2 cm) using a surgical scissor. About 200 µl of sterile PBS was placed on the center of the egg membrane (inner shell membrane) using a micropipette under the microscope (Zeiss Stemi 508 stereo zoom, Zeiss, Germany). The inner shell membrane was punctured carefully using a tweezer without injuring the underlying CAM to allow PBS to spread between the two membranes and separate them. This facilitated the easy removal of the inner shell membrane without injuring the CAM.

In order to inject the cells into the embryo, GFP-MDA-231 cells were suspended at a concentration of 5 × 10^6^ cells/ml in serum-free RPMI media. Ten microliters of the cell suspension (5 × 10^4^ cells) was taken in a microinjection needle (Glass Capillary Narishige, US) which was connected to a pneumatic microinjector (IM-11-2, Narishige) through a silicon tubing. The needle was used to pierce the CAM very carefully and inject the cells into the heart of the embryo. Then, the window in the eggshell was closed with a cellophane tape. For each experimental series, 5 controls were kept without injecting anything into the embryo; another 5 controls were used in which 10 µl of RPMI medium was injected, and the remaining 20 embryos were injected with GFP-MDA-231 cells to monitor the cell migration at different time frames ranging from 2 to 6 days after injection.

**FIGURE 2:**
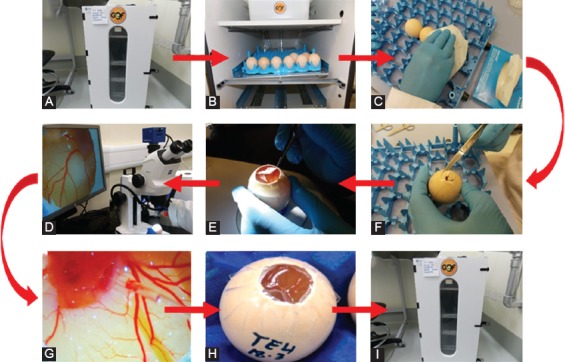
Steps of the injection process of green fluorescent protein (GFP)-MDA-231 cells in chicken embryo. (A and B) Incubation of chicken eggs; (C) surface sterilization of the eggs using tissue paper wetted with 70% ethanol; (D) making a hole in the eggshell; (E) removal of the egg membrane to expose the chorioallantoic membrane (CAM); (F) locating the injection site under a microscope; (G) injection of GFP-MDA-231 cells; (H) sealing of the shell window using cellophane tape; (I) incubation of the eggs.

Post injection of GFP-MDA-231 cells, the embryos were placed in the egg incubator by keeping the egg tray rack in a static setting (at 37°C, 70% humidity). The main steps of the cancer cell injection process are shown in Figure 2.

### Isolation of organs and processing tissue samples for microscopy

**FIGURE 3:**
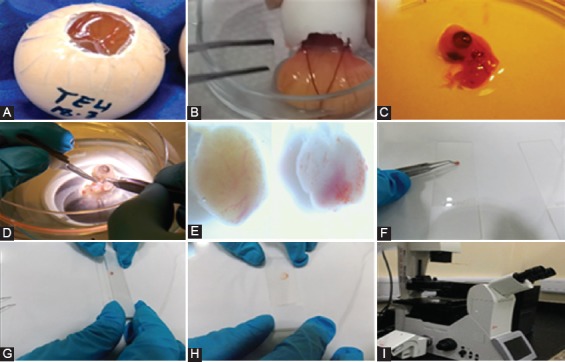
Isolation of organs from chicken embryo after different time points of injection of green fluorescent protein (GFP)-MDA-231 cells. (A) Chicken egg containing the embryo injected with cancer cells; (B) chicken embryo with extraembryonic fluids transferred to a Petri dish; (C) isolated chicken embryo; (D) dissection of the embryo to isolate various organs; (E) isolated embryonic organs; (F) transferring the parts of isolated organs into a clean microscopic glass slide; (G) arranging the parts of isolated organs between two slides; (H) pressing the organs between glass slides; (I) observing the slides under a fluorescent microscope.

For the microscopic analysis and quantification of the migrated cancer cells to various organs of chicken embryo, organs were isolated after different time points and observed under a fluorescent microscope, as shown in Figure 3.

#### Isolation of organs

The embryos were dissected at EDD-5, EDD-7, and EDD-9 to isolate various organs from the embryo which were at the 2^nd^, 4^th^, and 6^th^ day of post-injection, respectively. Cellophane tapes were removed from the eggs using blunt end tweezers, and the embryos were transferred individually to Petri dishes. In order to avoid cross-contamination, two separate sets of surgical tools were used, one set for dissecting controls and one set for dissecting GFP-MDA-231 injected embryos. Different wash containers filled with PBS were maintained to wash the tools before working on different embryos. Each isolated embryo was washed several times with PBS and transferred to another fresh Petri dish containing PBS. Embryos were dissected using tweezers and scissors to extract heart, brain, and liver; dissected organs were washed again individually in PBS. These organs were chosen for their ease of recognition and isolation. However, other organs or tissues can be isolated based on the specific needs of the research project.

### Observation of organs under a microscope

Fluorescent microscopy was used to detect migrated cells in various organs of the chicken embryo. GFP-MDA-231 cells can be excited by 488 nm laser light and optically detected at 510 nm using the GFP filter of the inverted fluorescent microscope (Leica DMi8). Repeated washing of organs was performed to remove any floating GFP-MDA-231 cells that might be present in body fluids. In order to visualize the migrated GFP-MDA-231 cells in the organs two different approaches were used, namely, direct observation (without any tissue preparation) and a novel simple approach using microscopic slides.

#### Direct observation of organs

Properly washed organs were transferred into the wells of 12-well plate and observed under the fluorescent microscope (Leica DMi8, 10× objective) with GFP filter to visualize the cells. Entire parts of each organ were analyzed to find and image the migrated GFP-MDA-231 cells, if any. Observed GFP positive cells were counted and tabulated.

#### Preparation of microscopic slides

Imaging of thick samples using fluorescence microscopy was a challenging task due to the high level of background noise and difficulty of getting images of cells which were deep inside the tissues. Thus, we used a novel and simple approach to make thinner microscopic specimens. For this, each isolated organ was minced into 2–4 pieces based on their size. Tissue pieces were placed between clean microscopic glass slides, which were placed on a flat surface (a table) and pressed by hand firmly for about 10 sec. The entire area of the glass slides (with the tissues) was observed under the fluorescent microscope (Leica DMi8, 10× objective) to detect and visualize the migrated GFP expressing cancer cells. Then, observed GFP positive cells were counted and tabulated.

## RESULTS

### Imaging of migrated cells in unprocessed tissue samples

**FIGURE 4:**
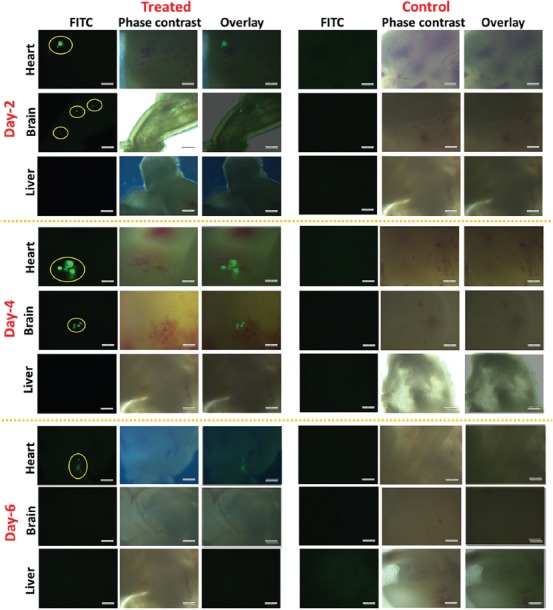
Green fluorescent protein (GFP)-MDA-231 cells detected in various organs of chicken embryo at days 2, 4, and 6 of post-injection (scale bar: 200 µm).

After the injection of transfected cancer cells into the heart of the embryo, they were dissected at days 2, 4, and 6 of injection. Images of the heart, brain, and liver were taken under a fluorescent microscope to confirm the presence of migrated cells. Pictures of GFP-MDA-231 cells in the heart, brain, and liver of intact organs of chicken embryo are shown in Figure 4 and Table 1. In many cases, GFP-MDA-231 cells were not clearly identifiable in the intact organs under the microscope because of the higher thickness of the samples for microscopic observation; this was especially apparent for the liver and brain. In general, heart tissues possessed the highest number of GFP expressing cells, which is consistent with it being the primary injection site. However, cells remained in colonies or aggregates, which made cell counting particularly difficult. On the other hand, the brain contained a smaller number of migrated GFP cells compared to the heart, while liver tissues showed no migrated GFP expressing cells. Since migrated cells were mostly in aggregates, data obtained from the direct imaging of organs cannot be properly quantified, which necessitates the use of the tissue sampling process described above, followed by microscopic analysis.

**TABLE 1:**
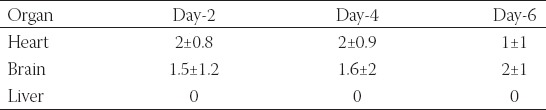
Migrated green fluorescent protein (GFP)-MDA-231 cells quantified using fluorescent images of organs isolated from chicken embryos at days 2, 4, and 6 of post-injection

### Imaging of migrated cells in glass slides containing manually pressed tissue samples

**FIGURE 5:**
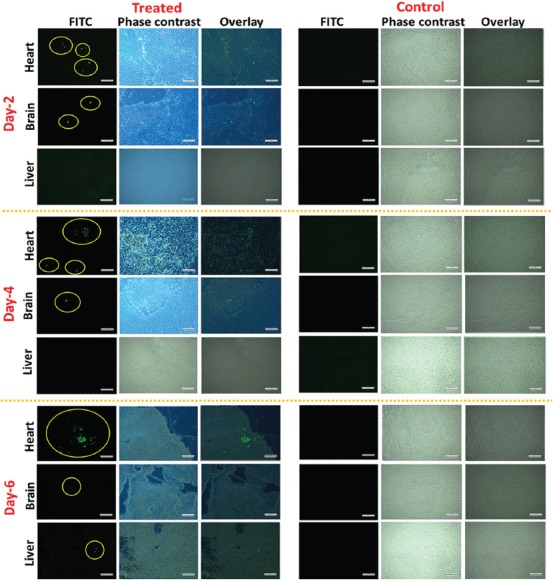
Green fluorescent protein (GFP)-MDA-231 cells detected in pressed samples of various organs of chicken embryos at days 2, 4, and 6 of post-injection (scale bar: 200 µm).

Due to the difficulties in obtaining consistent results from the direct imaging of isolated organs, we used the slide pressing technique to get thin specimens of tissues, which was very simple, fast, and efficient. After pressing the organs between microscopic slides, migrated GFP-MDA-231 cells were easily detectable. Moreover, quantification of migrated cells was more reliable and reproducible compared to directly imaged organs. Figure 5 and Table 2 show observed GFP-MDA-231 cells in the heart, brain, and liver of the chicken embryos. Unlike unprocessed organs, we did not observe aggregated GFP-MDA-231 cells, which facilitated the detection and quantification process. Thus, the number of migrated cells obtained in pressed organs was higher compared to that observed in the organs without slide pressing.

**TABLE 2:**
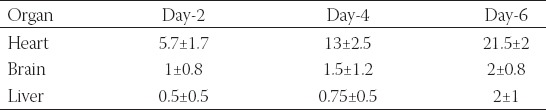
Migrated green fluorescent protein (GFP)-MDA-231 cells quantified from the fluorescent images of pressed organs which were isolated from the chicken embryos at days 2, 4, and 6 of post-injection

As observed, the heart showed the highest number of GFP-MDA-231 cells with the least number of GFP expressing cells on day 2 after injection. However, on days 4 and 6, there was an increase in the number of GFP expressing cells which can be ascribed to cell proliferation. The brain and liver tissues also showed a relatively similar trend; however, there was less migrated cells in comparison with the heart. Most importantly, using this method, we were able to detect migrated GFP-MDA-231 cells in the liver, which were not detectable in the intact organs.

## DISCUSSION

The use of chicken embryo-based metastasis models provides ample opportunities and yet harbors some challenges for qualitative and quantitative imaging and image analysis. On the other hand, histopathological analysis enables tracking of migrated cells into various organs [35]. However, such approaches are both time-consuming and laborious. Thus, we developed a simple method for studying cancer metastasis using chicken embryos and image analysis to detect migrated cancer cells in various organs. This simple and manual technique allows for quick recovery of quantitative data using tools that are readily available in any biological lab.

While other researchers described the use of chicken embryo models to study cancer metastasis [36], most of them rely on the CAM assay, which is considerably different from our model where we directly injected cancer cells into the heart of the embryo. In one such study, the CAM model was used to examine ovarian cancer cell invasion and metastasis to the posterior CAM and lungs of chicken embryos using various cells such as IGROV-1 [37], OVCAR-3, SKOV-3, and OV-90 [38]. Admittedly, while, this CAM model may mimic cell migration across peritoneum, nevertheless, it may not be an appropriate model for cell migration to other organs such as brain, liver, or lungs. The slide preparation technique used in this study was a very simple approach and allowed the quick quantification of migrated fluorescently labeled cancer cells to various organs of the embryo. Such a quick and accurate quantification of metastatic cells was not possible in earlier chicken embryo-based models [39].

Therefore, we believe that there are several advantages for our model over the existing *in vitro* and *in vivo* models of cancer metastasis, such as cost-effectiveness, speed of performing and obtaining results, as well as simplicity of experimental procedures. It also provides a more realistic and complex bio-microenvironment compared to *in vitro* models [40]. Most importantly, this model is highly flexible and can be adapted with minor modifications to suit a vast variety of applications. For instance, it can aid in understanding the mechanisms of cancer metastasis as well as the effect of certain inhibitors, drugs, or radiation on cancer metastasis using various cell types. In the earlier studies, using the chicken embryo CAM assay and *in vitro* cell culture, our group showed that cell-phone radio frequency can promote angiogenesis and cancer cell invasion [41]; additionally, the effect of cell-phone radio frequency on cancer cell metastasis can be verified using this model. Our *ex ovo* model also overcomes some of the limitations of widely-used zebrafish models [42]. In this regard, it is worth noting that most mammalian tumors proliferate at 37°C, which is also the optimum temperature to incubate the chicken embryos; unlike the zebrafish model which optimally grows at 31°C [43].

Moreover, this model avoids several ethical and regulatory complications since it uses chicken embryos of <10 days of incubation (EDD-10). In several countries, chicken embryos that are ≤14 days of incubation are not considered to be living organisms that can experience pain [44,45]; hence, ethical approvals are not required to perform experiments with chicken embryos ≤EDD-10. However, this may vary among different countries or depending on the regulations of individual institutions. On the other hand, the direct injection of cancer cells into the developing heart allows embryos to be analyzed at much earlier stages than other chicken embryo-based approaches, including CAM-based models.

Nevertheless, in spite of the numerous advantages of this model, several precautionary measures need to be taken, most specifically pertaining to the injection of cancer cells into the embryo. This critical process needs to be consistent and performed delicately in order to avoid premature death of the embryo and to obtain reliable results. In this study, eggs were observed every 24 h for signs of embryo discoloration. Those with color variations were opened by removing the cellophane tape, and dead eggs were counted, tabulated, and discarded. Comparing this between controls and cancer cell injected chicken embryos within 24–48 h of injection, we noticed that a relatively small number of embryos died after 24–48 h of injection (data not shown).

## CONCLUSION

Due to the growing importance of understanding cancer metastasis, various models are in developmental or testing stage to screen new genes and compounds that can prevent this fatal disease. Most importantly, focus on the components of tumor microenvironment, such as the extracellular matrix, microRNA profiles, pH, fluid flow, and interstitial pressure with regard to their role in cancer metastasis has opened new avenues in metastatic cancer research and therapy. Therefore, the inclusion of these components in metastasis models is necessary to mimic the actual tumor microenvironment for better screening of drugs as well as oncogenic initiatives. Our study suggests that chicken embryo-based *in ovo* metastasis models could be a promising strategy for mimicking such an environment. In this study, we were able to demonstrate that fluorescently labeled MDA-MB-231 cancer cells migrate to various organs such as brain and liver, where they can potentially proliferate for a period of up to 6 days. The use of GFP expressing cancer cells allows easy detection and quantification of such migrated cancer cells. Moreover, the application of our slide pressing approach improves the quantification and detection of migrated cancer cells into various tissues and organs. This minimizes the time and effort required compared to conventional histopathological analysis. Thus, the combination of these approaches with further improvements, such as the use of multiple cell lines and anti-metastatic agents, can open new horizons in cancer biology and pharmaceutical research.

## References

[1] Seyfried TN, Huysentruyt LC (2013). On the origin of cancer metastasis. Crit Rev Oncog.

[2] Valastyan S, Weinberg RA (2011). Tumor metastasis:Molecular insights and evolving paradigms. Cell.

[3] Krušlin B, Ulamec M, Tomas D (2015). Prostate cancer stroma:An important factor in cancer growth and progression. Bosn J Basic Med Sci.

[4] Guan X (2015). Cancer metastases:Challenges and opportunities. Acta Pharm Sin B.

[5] Zeeshan R, Mutahir Z (2017). Cancer metastasis tricks of the trade. Bosn J Basic Med Sci.

[6] Bhowmik A, Khan R, Ghosh MK (2015). Blood brain barrier:A challenge for effectual therapy of brain tumors. Biomed Res Int.

[7] Langley RR, Fidler IJ (2007). Tumor cell-organ microenvironment interactions in the pathogenesis of cancer metastasis. Endocr Rev.

[8] Debeir OD, Decaestecker C, Adanja I, Kiss R (2008). Models of cancer cell migration and cellular imaging and analysis In:Anja Lambrechts, Christophe Ampe, editors. The Motile Actin System in Health and Disease. Thiruvananthapuram Transworld Research Signpost.

[9] Malandrino A, Kamm RD, Moeendarbary E (2018). *In vitro* modeling of mechanics in cancer metastasis. ACS Biomater Sci Eng.

[10] Khanna C, Hunter K (2005). Modeling metastasis *in vivo*. Carcinogenesis.

[11] Gandalovičová A, Rosel D, Fernandes M, Veselý P, Heneberg P, Čermák V (2017). Migrastatics-anti-metastatic and anti-invasion drugs:Promises and challenges. Trends Cancer.

[12] Anderson RL, Balasas T, Callaghan J, Coombes RC, Evans J, Hall JA (2019). A framework for the development of effective anti-metastatic agents. Nat Rev Clin Oncol.

[13] Katt ME, Placone AL, Wong AD, Xu ZS, Searson PC (2016). *In vitro* tumor models:Advantages, disadvantages, variables, and selecting the right platform. Front Bioeng Biotechnol.

[14] Hulkower KI, Herber RL (2011). Cell migration and invasion assays as tools for drug discovery. Pharmaceutics.

[15] Menyhárt O, Harami-Papp H, Sukumar S, Schäfer R, Magnani L, de Barrios O (2016). Guidelines for the selection of functional assays to evaluate the hallmarks of cancer. Biochim Biophys Acta.

[16] Langhans SA (2018). Three-dimensional *in vitro* cell culture models in drug discovery and drug repositioning. Front Pharmacol.

[17] Liu M, Scanlon CS, Banerjee R, Russo N, Inglehart RC, Willis AL (2013). The histone methyltransferase EZH2 mediates tumor progression on the chick chorioallantoic membrane assay, a novel model of head and neck squamous cell carcinoma. Transl Oncol.

[18] Ferdowsian HR, Beck N (2011). Ethical and scientific considerations regarding animal testing and research. PLoS One.

[19] Gómez-Cuadrado L, Tracey N, Ma R, Qian B, Brunton VG (2017). Mouse models of metastasis:Progress and prospects. Dis Model Mech.

[20] Murphy JB (1913). Transplantability of tissues to the embryo of foreign species:Its bearing on questions of tissue specificity and tumor immunity. J Exp Med.

[21] Bachnou N, Saule S, Dieterlen-Lievre F (1996). An identical effect mediated by thyroid deficiency or oncogene v-erbA in the chick embryo. Int J Dev Biol.

[22] Ossowski L, Reich E (1980). Experimental model for quantitative study of metastasis. Cancer Res.

[23] Subauste MC, Kupriyanova TA, Conn EM, Ardi VC, Quigley JP, Deryugina EI (2009). Evaluation of metastatic and angiogenic potentials of human colon carcinoma cells in chick embryo model systems. Clin Exp Metastasis.

[24] Vranic S, Marchiò C, Castellano I, Botta C, Scalzo MS, Bender RP (2015). Immunohistochemical and molecular profiling of histologically defined apocrine carcinomas of the breast. Hum Pathol.

[25] Roganovic D, Djilas D, Vujnovic S, Pavic D, Stojanov D (2015). Breast MRI, digital mammography and breast tomosynthesis:Comparison of three methods for early detection of breast cancer. Bosn J Basic Med Sci.

[26] Kaijzel EL, Snoeks TJ, Buijs JT, van der Pluijm G, Löwik CW (2009). Multimodal imaging and treatment of bone metastasis. Clin Exp Metastasis.

[27] Progatzky F, Dallman MJ, Lo Celso C (2013). From seeing to believing:Labelling strategies for *in vivo* cell-tracking experiments. Interface Focus.

[28] Pichorner A, Sack U, Kobelt D, Kelch I, Arlt F, Smith J (2012). *In vivo* imaging of colorectal cancer growth and metastasis by targeting MACC1 with shRNA in xenografted mice. Clin Exp Metastasis.

[29] Chudakov DM, Matz MV, Lukyanov S, Lukyanov KA (2010). Fluorescent proteins and their applications in imaging living cells and tissues. Physiol Rev.

[30] Khan KH (2013). Gene expression in mammalian cells and its applications. Adv Pharm Bull.

[31] Langenau DM, Ferrando AA, Traver D, Kutok JL, Hezel JP, Kanki JP (2004). *In vivo* tracking of T cell development, ablation, and engraftment in transgenic zebrafish. Proc Natl Acad Sci U S A.

[32] Kasiotis KM, Magiatis P, Pratsinis H, Skaltsounis A, Abadji V, Charalambous A (2001). Synthesis and biological evaluation of novel daunorubicin-estrogen conjugates. Steroids.

[33] Goggins E, Kakkad S, Mironchik Y, Jacob D, Wildes F, Krishnamachary B (2018). Hypoxia inducible factors modify collagen I fibers in MDA-MB-231 triple negative breast cancer xenografts. Neoplasia.

[34] Longo PA, Kavran JM, Kim MS, Leahy DJ (2014). Single cell cloning of a stable mammalian cell line. Methods Enzymol.

[35] Tomura M, Hata A, Matsuoka S, Shand FH, Nakanishi Y, Ikebuchi R (2014). Tracking and quantification of dendritic cell migration and antigen trafficking between the skin and lymph nodes. Sci Rep.

[36] Deryugina EI, Quigley JP (2008). Chick embryo chorioallantoic membrane model systems to study and visualize human tumor cell metastasis. Histochem Cell Biol.

[37] Chang HL, Pieretti-Vanmarcke R, Nicolaou F, Li X, Wei X, MacLaughlin DT (2011). Mullerian inhibiting substance inhibits invasion and migration of epithelial cancer cell lines. Gynecol Oncol.

[38] Lokman NA, Elder AS, Ricciardelli C, Oehler MK (2012). Chick chorioallantoic membrane (CAM) assay as an *in vivo* model to study the effect of newly identified molecules on ovarian cancer invasion and metastasis. Int J Mol Sci.

[39] Bobek V, Plachy J, Pinterova D, Kolostova K, Boubelik M, Jiang P (2004). Development of a green fluorescent protein metastatic-cancer chick-embryo drug-screen model. Clin Exp Metastasis.

[40] Hendrix MJ, Seftor EA, Seftor RE, Kasemeier-Kulesa J, Kulesa PM, Postovit LM (2007). Reprogramming metastatic tumour cells with embryonic microenvironments. Nat Rev Cancer.

[41] Alahmad YM, Aljaber M, Saleh AI, Yalcin HC, Aboulkassim T, Yasmeen A (2018). Effect of cell-phone radiofrequency on angiogenesis and cell invasion in human head and neck cancer cells. Head Neck.

[42] Tat J, Liu M, Wen XY (2013). Zebrafish cancer and metastasis models for *in vivo* drug discovery. Drug Discov Today Technol.

[43] Sfakianakis DG, Leris I, Kentouri M (2011). Effect of developmental temperature on swimming performance of zebrafish (*Danio rerio*) juveniles. Environ Biol Fishes.

[44] Ribatti D (2016). The chick embryo chorioallantoic membrane (CAM) A multifaceted experimental model. Mech Dev.

[45] Dupertuis YM, Delie F, Cohen M, Pichard C (2015). *In ovo* method for evaluating the effect of nutritional therapies on tumor development, growth and vascularization. Clin Nutr Exp.

